# Supplementary material for: Tumor purity-related genes for predicting the prognosis and drug sensitivity of DLBCL patients
Source: eLife. 2024 Jul 9;13:RP92841. doi: 10.7554/eLife.92841 (PMC11233133; doi:10.7554/eLife.92841)
Supplement: Supplementary file 1. [file elife-92841-supp1.docx]

**Supplementary File 1 The Clinicopathological Characteristics of CHCAMS Cohort**

| **Characteristics** | **n(%)** |
| --- | --- |
| **Sex** |  |
| male | 106(55.8%) |
| female | 84(44.2%) |
| **Age** (53.85±1.14^a^) |  |
| ≤60 | 115(60.5%) |
| ＞60 | 75(39.5%) |
| **Intra or Extra LN^b^** |  |
| intra | 82(43.2%) |
| extra | 108(56.8%) |
| **Position** |  |
| neck LN^b^ | 59(31.1%) |
| testis | 26(13.7%) |
| colon | 25(13.2%) |
| groin LN^b^ | 14(7.3%) |
| others | 66(34.7%) |
| **VCAN-H Score** (cutoff =275.42, 233.37±4.60^a^) |  |
| Low | 127(66.8%) |
| High | 63(33.2%) |
| **CD3G+ T cells-ratio** (%, cutoff = 2.5%, 14.25±1.32^a^) |  |
| Low | 61(32.1%) |
| High | 129(67.9%) |
| **C1QB-H Score** (cutoff = 82.41, 65.98±3.01^a^) |  |
| Low | 117(61.6%) |
| High | 73(38.4%) |
| **CD68+ Mφ -ratio** (%, cutoff = 18.6%, 17.75±1.05^a^) |  |
| Low | 125(65.8%) |
| High | 65(34.2%) |
| **CD4+ T cells-ratio** (%, cutoff = 0.13%, 0.68±0.20^a^) |  |
| Low | 132(69.5%) |
| High | 58(30.5%) |
| **CD8+ T cells-ratio** (%, cutoff = 0.375%, 6.69±0.56^a^) |  |
| Low | 24(12.6%) |
| High | 166(87.4%) |
|  |  |

a. mean±SEM, b. Lymph node
