# Supplementary material for: Tumor purity-related genes for predicting the prognosis and drug sensitivity of DLBCL patients
Source: eLife. 2024 Jul 9;13:RP92841. doi: 10.7554/eLife.92841 (PMC11233133; doi:10.7554/eLife.92841)
Supplement: Supplementary file 2. [file elife-92841-supp2.docx]

**Supplementary File 2 The prediction of drug sensitivity**

| **Drugs** | **GSE53786 Sensitivity Score (Mean ± SEM)** | | | **GSE32918 Sensitivity Score (Mean ± SEM)** | | |
| --- | --- | --- | --- | --- | --- | --- |
|  | Low | High | P value^1^ | Low | High | P value^1^ |
| **Carmustine** | 486.997±11.783 | 421.590±10.472 | 0.0000385 | 474.022±8.196 | 432.643±6.370 | 0.000086 |
| **Cytarabine** | 6.668±0.435 | 5.361±0.307 | 0.028 | 6.894±0.361 | 5.654±0.352 | 0.0000353 |
| **Oxaliplatin** | 52.990±3.395 | 37.582±1.864 | 9.19×10^-6^ | 48.933±1.720 | 40.808±1.666 | 4.35×10^-6^ |
| **Vincristine** | 0.203±0.014 | 0.146±0.008 | 0.0005 | 0.219±0.026 | 0.223±0.031 | 0.0047 |
| **Vorinostat** | 4.509±0.131 | 4.066±0.120 | 0.0138 | 4.618±0.091 | 3.924±0.056 | 7.09×10^-9^ |
| **Afuresertib** | 13.022±0.581 | 13.420±0.603 | 0.5932 | 13.847±0.362 | 12.380±0.338 | 0.0037 |
| **Bortezomib** | 0.008±0.0002 | 0.007±0.0002 | 0.0355 | 0.0081±0.0002 | 0.0076±0.0002 | 0.0035 |
| **Ibrutinib** | 97.082±3.271 | 91.220±3.254 | 0.2068 | 98.641±2.116 | 87.625±1.638 | 0.0000836 |
| **Tamoxifen** | 37.392±1.129 | 34.720±0.904 | 0.1536 | 37.358±0.627 | 34.545±0.669 | 0.0002 |
| **OTX015** | 13.468±0.672 | 11.710±0.485 | 0.0905 | 12.439±0.351 | 12.818±0.590 | 0.1642 |
| **Cyclophosphamide** | 177.152±3.594 | 170.524±3.350 | 0.203 | 176.726±2.868 | 173.443±3.273 | 0.1019 |
| **Dinaciclib** | 0.0607±0.0014 | 0.0606±0.0014 | 0.8712 | 0.061±0.001 | 0.065±0.003 | 0.5872 |
| **Buparlisib** | 2.590±0.049 | 2.617±0.057 | 0.7721 | 2.615±0.039 | 2.643±0.057 | 0.2177 |
| **Alisertib** | 7.352±0.332 | 6.855±0.354 | 0.1566 | 7.284±0.222 | 6.947±0.247 | 0.1359 |
| **Gemcitabine** | 0.696±0.111 | 0.595±0.062 | 0.2391 | 0.732±0.104 | 0.818±0.153 | 0.1453 |

1. Wilcoxon rank sum test, P value < 0.05 is regarded as statistically significant.
